# Associations between accelerometer-assessed occupational and leisure-time sitting and self-reported cognitive dysfunction in Swedish middle-aged adults: a cross-sectional analysis from the SCAPIS study

**DOI:** 10.1186/s12889-026-29397-4

**Published:** 2026-09-10

**Authors:** Frida Bergman, Carl-Johan Boraxbekk, Pasan Hettiarachchi, Magnus Svartengren, Patrik Wennberg, Melony Fortuin-de Smidt

**Affiliations:** 1 Department of Public Health and Clinical Medicine, Umeå University, Umeå, Sweden; 2 Department of Health, Education and Technology, Luleå University of Technology, Luleå, Sweden; 3 Institute for Clinical Medicine, Faculty of Medical and Health Sciences, University of Copenhagen, Copenhagen, Denmark; 4 Institute of Sports Medicine Copenhagen (ISMC) and Department of Neurology, Copenhagen University Hospital Bispebjerg, Copenhagen, Denmark; 5 Occupational and Environmental Medicine, Department of Medical Sciences, Uppsala University, Uppsala, Sweden

**Keywords:** Accelerometry, Cognition, Sedentary behavior, Sedentary domains

## Abstract

**Background:**

The relationship between sedentary behavior and cognitive health remains insufficiently understood. The aim of this study was to examine associations between occupational and leisure-time sitting and self-reported cognitive dysfunction.

**Methods:**

Data from men and women, aged 50–64 years old, participating in the Umeå site of the cross-sectional Swedish CArdioPulmonary bioImage Study (SCAPIS) was used. Occupational and leisure-time sitting were measured using a thigh-worn accelerometer over seven days. Work hours were reported in a diary. Cognitive dysfunction was measured using the Cognitive Dysfunction Questionnaire. A Hurdle model was used to firstly assess the association of domain-specific sitting time with any cognitive dysfunction (logistic regression) and secondly with the severity of cognitive dysfunction (beta-binomial regression). Analyses were adjusted for sex, age, educational level, moderate- to vigorous physical activity, sleep duration, risky alcohol intake and emotional health limitations.

**Results:**

A total of 1559 participants were included. In the fully adjusted model, each additional hour of occupational sitting was associated with 8% lower odds of reporting cognitive dysfunction (odds ratio (OR) 0.92, 95% confidence interval (CI) 0.87–0.98), while each additional hour of leisure-time sitting was associated with 12% higher odds (OR 1.12, 95% CI 1.03–1.21). Neither occupational nor leisure-time sitting was significantly associated with the severity of cognitive dysfunction among individuals reporting any dysfunction.

**Conclusions:**

Domain-specific sitting time was associated with the occurrence, but not the severity, of self-reported cognitive dysfunction in middle-aged adults. The contrasting associations observed for occupational and leisure-time sitting suggest that the context of sedentary behavior may be relevant for cognitive health. Future longitudinal studies using device-based measurements are needed to clarify causality and to explore the potential role of cognitively active and passive sedentary behaviors.

## Background

With aging populations, the prevalence of cognitive decline and dementia is steadily increasing, with an estimated increase in dementia cases from 57.4 million in 2019 to 152.8 million in 2050 globally [1]. Cognitive dysfunction can range from mild impairments with minimal influence on daily life to severe forms such as dementia. Subjective cognitive decline refers to an individual’s experience of persistent deterioration in cognitive capacity compared to their previous normal state, unrelated to any acute event, despite normal performance on standardized cognitive tests [2]. Subjective cognitive decline has been associated with an increased risk of future development of mild cognitive impairment and dementia [3]. These associations have been observed to be moderated by age, where people under the age of 70 with subjective cognitive decline had a greater risk of developing mild cognitive impairments compared to those over the age of 70 [3]. Therefore, improving our understanding of risk factors associated with subjective cognitive decline in middle-aged people is of great importance for early prevention strategies.

Different physical behaviors are often targeted as prevention strategies for health outcomes, including cognitive function and brain health. One of the recommended prevention approaches for dementia is the encouragement of physical activity throughout the life course [4]. For example, midlife physical activity has been associated with a lower risk for all-cause dementia and Alzheimer’s disease in follow-ups longer than 20 years [5]. Further, associations between sedentary behavior and cognitive functions have also been studied, but the evidence is less profound. A systematic review and meta-analysis on middle-aged and older adults observed no association between higher total sedentary time and worse cognitive function, but large heterogeneity existed between the studies [6]. However, in sub-analyses of studies using devices to measure sedentary behavior, a higher sedentary time was significantly associated with worse cognitive function, specifically for global cognitive function and processing speed. Contrastingly, in sub-analyses of studies using self-reported measurements of sedentary behavior, a higher sedentary time was significantly associated with better cognitive function, specifically for processing speed [6]. These findings highlight the need for studies using more objective measurements, such as accelerometer-based measurements, since relying solely on self-reported sedentary behavior may increase the risk of bias with an underestimation of 2–3 h in time spent sitting in general [7]. Specifically, thigh-worn accelerometers can measure sedentary behavior with a high validity [8].

Several mechanisms may underlie the association between sedentary behavior and cognitive function. Prolonged sedentary behavior has been linked to metabolic dysfunction, systemic inflammation, impaired growth factor signaling, and cerebrovascular and neuroendocrine dysregulation, which may in turn contribute to structural and functional changes in the brain associated with cognitive decline [9, 10]. These pathways may act independently or interactively and may also be influenced by the broader context in which sedentary behavior occurs, including whether sitting takes place during work or leisure time. Previous studies have indicated that the domain in which sitting occurs might be of importance for the associations with cognitive function [11, 12]. Findings from one previous study indicated that occupational sitting was associated with better cognitive function, while leisure-time sitting showed no significant association [11]. However, these studies have analyzed self-reported occupational and leisure-time sedentary behaviors [11, 12]. Hence, domain-specific effects need to be explored further using thigh-accelerometer-based measurements of sedentary behaviors during both work and leisure-time [6].

The aim of this study was to investigate the associations between device-measured occupational and leisure-time sitting, measured using thigh-worn accelerometers, and subjective cognitive dysfunction in Swedish middle-aged adults.

## Method

### Study design

In this study, data from the cross-sectional Swedish CArdioPulmonary bioImage Study (SCAPIS) was used. The SCAPIS study protocol has been described in detail elsewhere [13]. Briefly, in SCAPIS, a random sample of men and women aged 50–64 years of age from the general population were invited for participation. Data were collected at six different university hospitals in Sweden, and 30,154 participants were included. Core measurements were performed at all SCAPIS study sites according to a standardized protocol. At each study site, local researchers could also add measurements based on their research interest. Measurements with thigh-worn accelerometers were performed at the Umeå, Uppsala and Gothenburg SCAPIS sites. In Umeå, cognitive dysfunction was measured using the Cognitive Dysfunction Questionnaire (CDQ). In line with the research questions in this study, only data from the Umeå SCAPIS site was used (*n* = 2507).

**Fig. 1:**
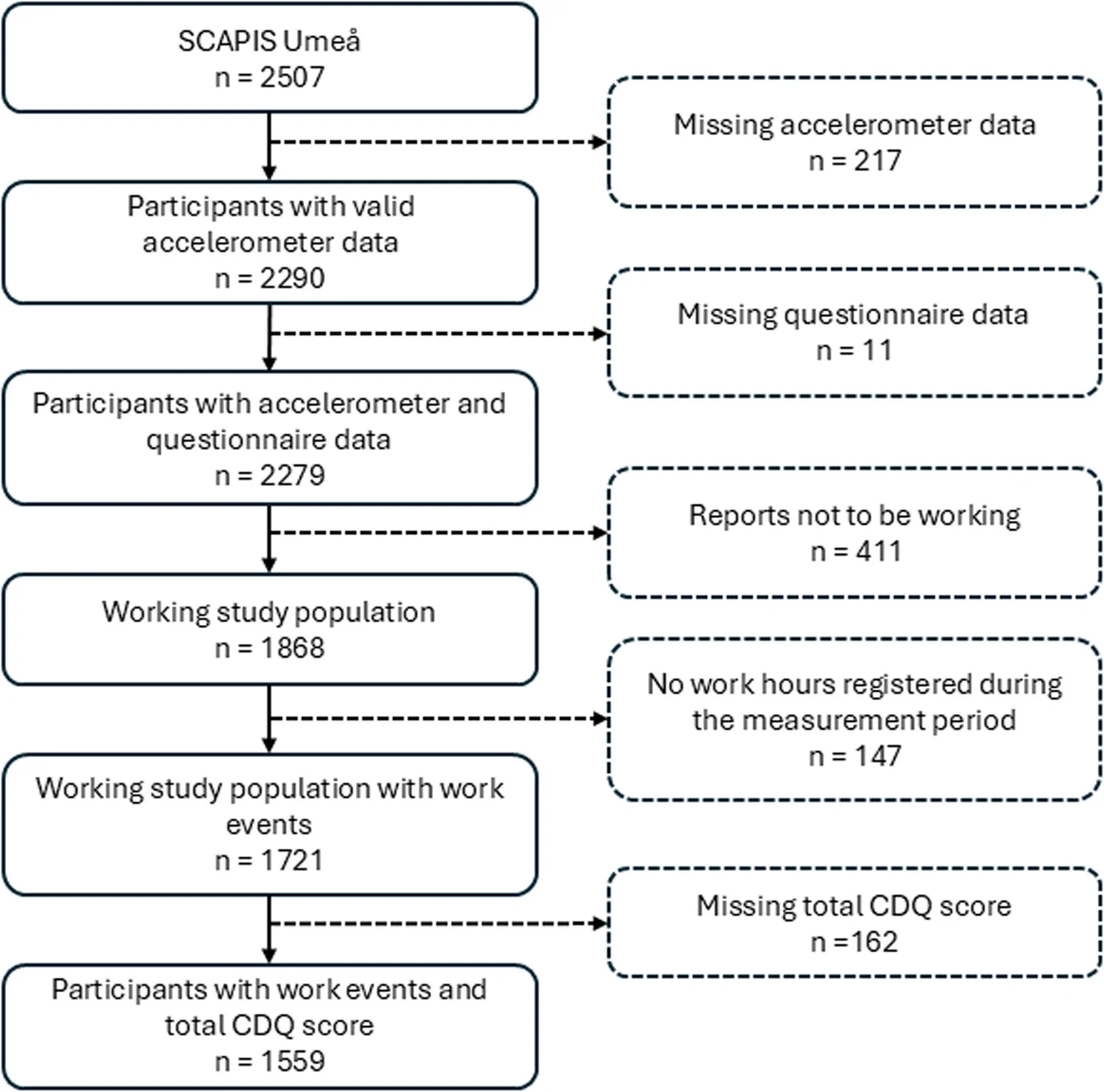
Flow chart of the study population. SCAPIS = Swedish CArdioPulmonary bioImage Study. CDQ = Cognitive Dysfunction Questionnaire

The inclusion criterium for SCAPIS was the ability to understand written and spoken Swedish for informed consent [13]. For our study, only those who reported to be working, who had working events reported during the measurement period, and who had complete data from the thigh-accelerometers and on the CDQ were included in the analyses. A total of 1559 participants had complete data on the CDQ, reported to be working and had working hours reported during the week of measurements and were thus included in the analyses (Fig. 1).

### Measurements

#### Cognitive dysfunction

In the SCAPIS Umeå cohort, subjective cognitive dysfunction was measured using the CDQ, previously developed by Vestergren et al. [14, 15]. In the CDQ, the participants were asked to report how often they have experienced cognitive failures during situations in their everyday life in the last year. The questionnaire consists of 20 items with answers given on a Likert scale, including the alternatives “Very seldom”, “Seldom”, “Sometimes”, “Often”, and “Very often”. Scores were assigned to each question, ranging between 1 (“Very seldom”) and 5 (“Very often”). Sub-scores, ranging between 4 and 20, were calculated for five different cognitive domains (procedural actions, semantic word knowledge, face recognition, temporal orientation, spatial navigation) and a total score of general cognitive function was calculated, ranging between 20 and 100. A score of 20 points indicates no dysfunction, and a higher score indicates a higher cognitive dysfunction. The final version of the CDQ can be found in Appendix A in Vestergren et al. [15]. Participants not responding to and/or giving multiple answers to a specific question in the CDQ did not receive a total cognitive score. The CDQ has been shown to have an acceptable construct and discriminant validity and to be psychometrically sound [14, 15].

#### Thigh accelerometer measurements

At the SCAPIS Umeå site, sedentary behavior, physical activity, and sleep were measured using the thigh-worn activPAL3 Micro accelerometer (PAL Technologies, Glasgow, Scotland, United Kingdom). At the first study visit, a research nurse attached the activPAL device to the front central right thigh of the participant. The participants were instructed to wear the device 24 h a day for seven consecutive days, only to be removed during water-based activities and in case of any discomfort. The device was returned to the study center on the next study visit or at any other convenient time. While wearing the device, participants were asked to record their workdays and work hours in a diary. The activPAL3 Micro accelerometer recorded raw triaxial accelerometer data at a sampling interval of 20 Hz, a dynamic range of +/- 2 g, and a resolution of 8-bit. The resulting raw accelerometer data was then processed by ActiPASS software (version 2024.06.3) [16]. ActiPASS is a brand agnostic software tool capable of processing raw data from thigh-worn accelerometers which has now being widely used for assessing physical and sedentary behaviours from free living accelerometer data [17–19]. ActiPASS, using validated and open algorithms [20–22], first generates a time-series of postures/activities and cadence (when applicable) at 1 s epoch (while the effective epoch length may vary depending on the posture and particular posture detection algorithm). This resulting 1 s epoch time series of postures/activities is then further processed by ActiPASS to generate variables containing duration of each posture/activity as minutes per 24-hour day.

Days with less than 20 h of non-wear, with less than 30 s of walking and more than 30 min of time in “other” behaviors (indicating an orientation issue or extreme individual calibration problem), with sensor errors, days that only consisted of sleep, or two or more consecutive days without a sleep interval were excluded from the analyses. A day was defined as the 24-hour time-period between 00:00 h to 00:00 h.

Sitting, moderate- to vigorous physical activity (MVPA) and sleep were classified as follows [20–25]. Occupational and leisure-time sitting was calculated as the mean hours in sitting and lying outside the periods flagged as sleep intervals and as total non-sleeping time during sleep intervals. MVPA was calculated as the total sum of hours spent in walking with cadence ≥ 100 steps/minute, running, cycling, inclined stepping, and unclassified behaviors with cadence ≥ 100/min during leisure-time. Sleep duration was calculated as the mean hours of sleep during the 24-hour day. All variables from the accelerometer data were calculated as mean or sum per participant over the valid measurement days.

#### Questionnaire data

From the SCAPIS questionnaire, information regarding the participants age, sex, and educational level, was used. Educational level was grouped as primary (no or primary degree education), secondary and tertiary educational level. Risky alcohol intake was defined as scoring 8 points or higher on the Alcohol Use Disorders Identification Test [26].

Emotional health limitations were assessed by the question “During the past 4 weeks, have you had any of the following problems with your work or other regular daily activities as a result of any emotional problems (such as feeling depressed or anxious)”, adapted from the Swedish SF-36 questionnaire [27–29]. The participants responded to two sub-questions: “Accomplished less than you would like?” and “Didn´t do work or other activities as carefully as usual”. The available answers were “Yes” or “No”. Emotional health limitations were classified as “Yes” if an individual had responded “Yes” to any or both questions.

### Statistical analysis

Descriptive data was reported as mean values with standard deviation for normally distributed data, as median values with interquartile range for non-normally distributed data, or as counts with percentage for categorical data.

**Fig. 2:**
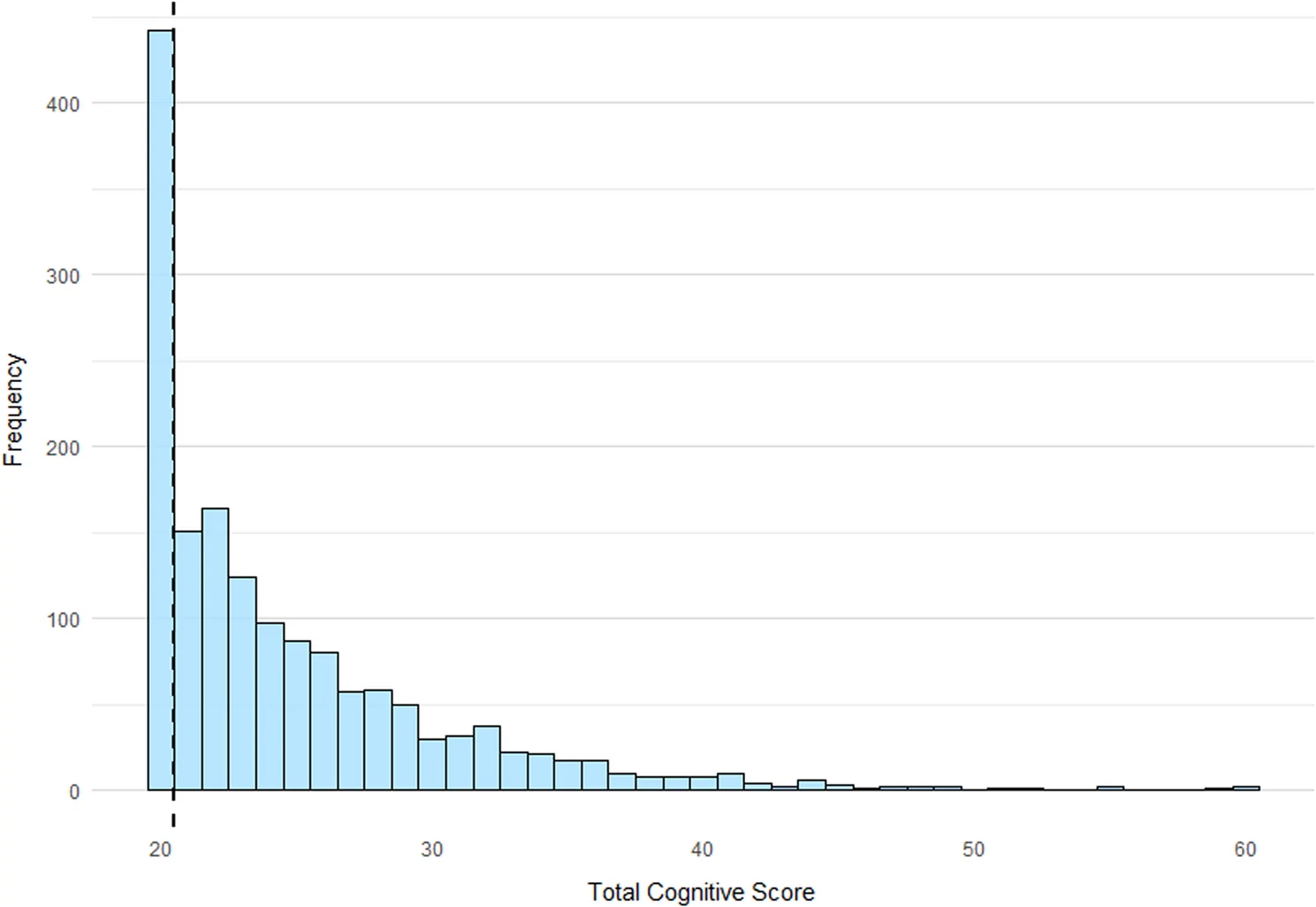
Distribution of the total cognitive score (*n* = 1559) from the Cognitive Dysfunction Questionnaire (CDQ). A higher score indicates a higher self-reported cognitive dysfunction. The vertical line indicates the border at 20 points of the CDQ, used as “the hurdle” in the statistical analysis

As the dependent variable was bounded between 20 and 100 points and very skewed to the right with a large proportion of participants having a CDQ score of 20 points (Fig. 2), a two-parted Hurdle-model was fitted. In a Hurdle model, the probability of the occurrence of the outcome is separated from the severity of the outcome among observations that have a value above (i.e. who have crossed) the hurdle. These types of models are thus suitable for data where a large part of the observations is stacked at one single point, commonly seen in e.g. psychological research [30]. In our analysis, CDQ scores were classified as 20 (no cognitive dysfunction) versus ≥ 21 (presence of cognitive dysfunction). This threshold was chosen based on the scoring structure of the CDQ, in which 20 points indicate an absence of dysfunction and any score above 20 indicates some degree of cognitive dysfunction, with higher scores indicates greater severity.

In our analysis, a logistic regression model was first fitted to determine the probability of reporting any level of cognitive dysfunction (i.e. whether the hurdle was crossed or not), with results reported as odds ratios (OR) with 95% confidence intervals (CI). Thereafter, associations between occupational and leisure-time sitting and the severity of cognitive dysfunction among participants with a CDQ score of 21 or higher (i.e. those who have crossed the hurdle) were modelled using a regression model with beta-binomial distribution and a logit link. The results from the second part were presented on the logit scale as beta-values with standard errors (SE).

Total CDQ score was used as the dependent variable. Multiple models with varying degrees of adjustment were fitted for both occupational and leisure-time sitting (mean hours/day): Model 1 – adjusted for age, sex, and educational level, Model 2 - in addition to model 1 variables further adjusted for total time spent in MVPA at leisure, mean hours of daily sleep duration, risky alcohol intake, and emotional health limitations [31–34], Model 3 – in addition to model 2 variables further included mutual adjustment for both work- and leisure-time sitting (Fig. 2).

The variables educational level, risky alcohol intake and emotional health limitations contained missing values (Table 1). Multiple imputation of missing independent variables was performed, using five imputed datasets and estimates were combined using Rubin´s rules.

Model assumptions were evaluated using residual diagnostics, multicollinearity checks, and dispersion inspections in the second part of the model. No evidence of severe misspecifications was observed.

Statistical analyses were performed using R version 4.4.1 [35] and RStudio v.2025.05.1.513 [36] using the libraries data.table v.1.17.8 [37], stringr v.1.5.2 [38], ggplot2 v.4.0.0 [39], writexlv.1.5.1 [40], readxl v.1.4.5 [41], vtable v.1.4.8 [42], glmmTMB [43, 44] and mice [45].

## Results

### Descriptive data

The characteristics of the study population are presented in Table 1. The participants had a median of six valid measurements days (interquartile range (IQR) 1) and four workdays (IQR 2). The mean valid duration was 8.5 h for worktime and 10.8 h for awake leisure-time. A total of 442 participants had 20 points on the CDQ, indicating no cognitive dysfunction, and 1117 participants had 21 points or higher on the CDQ, indicating cognitive dysfunction of different severity (Fig. 2).

**Table 1:** Characteristics of the included participants

Variable	Study population (*n* = 1559)
Age	56.9 (4.03)
Women (n, %)	808 (52)
Educational level (n, %)	
* Primary*	104 (6.7)
* Secondary*	752 (48.2)
* Tertiary*	702 (45.0)
* Missing*	1 (0.1)
Risky alcohol intake (n, %)	
* Yes*	100 (6.4)
* No*	1419 (91.0)
* Missing*	40 (2.6)
Emotional health limitations (n, %)	
* Yes*	219 (14.0)
* No*	1321 (84.7)
* Missing*	19 (1.2)
Cognitive Dysfunction Questionnaire (Median, IQR)	23 (7)
Mean sitting at work (h/day)	4.9 (1.9)
Mean sitting at leisure (h/day)	6.7 (1.5)
Total sum of MVPA at leisure (total h)	5.7 (2.6)
Mean sleep duration (h/day)	7.4 (1.1)

Data are presented as mean (SD) if not reported otherwise

*IQR* Interquartile Range, *MVPA* Moderate to Vigorous Physical Activity

### Main analyses

**Fig. 3:**
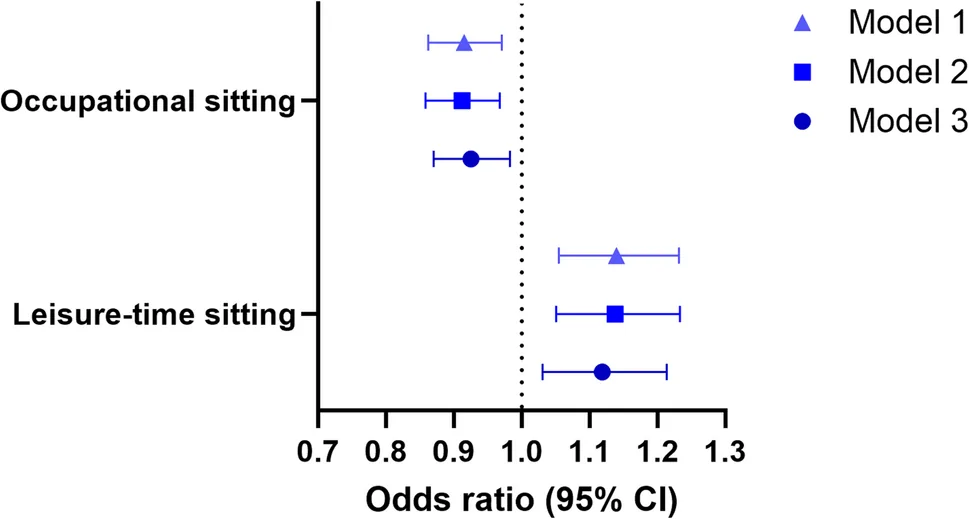
Associations of occupational and leisure-time sitting with having any level of cognitive dysfunction (i.e. CDQ score > 20) (*n* = 1559). Model 1 was adjusted for age, sex, and educational level. Model 2 was further adjusted for leisure-time moderate- to vigorous physical activity, sleep duration, risky alcohol intake, and emotional health limitations. Model 3 was further mutually adjusted for occupational and leisure-time sitting

In the logistic regression analysis, each hour increase in occupational sitting was associated with 8% lower odds of having any level of self-reported cognitive dysfunction (model 1: OR = 0.92, 95% CI 0.86–0.97, *p* = 0.003). This association remained significant when further adjusting for leisure-time MVPA, sleep duration, risky alcohol intake, and emotional health limitations (model 2: OR = 0.91, 95% CI 0.86–0.97, *p* = 0.003), and when mutually adjusting for leisure-time sitting (model 3: OR = 0.92, 95% CI 0.87–0.98 *p* = 0.013) (Fig. 3). Each hour increase in leisure-time sitting was significantly associated with 14% higher odds of having any level of cognitive dysfunction (model 1: OR = 1.14, 95% CI 1.05–1.23, *p* < 0.001). This association remained significant when further adjusting for leisure-time MVPA, sleep duration, risky alcohol intake, and emotional health limitations (model 2: OR = 1.14, 95% CI 1.05–1.23, *p* = 0.002), and when mutually adjusting for occupational sitting (model 3: OR = 1.12, 95% CI 1.03–1.21 *p* = 0.007) (Fig. 3).

In the beta-binomial regression model, among those with CDQ score of 21 points and higher, no significant associations between occupational or leisure-time sitting and severity of cognitive dysfunction were observed in any of the models (Table 2).

A sensitivity analysis restricted to complete cases yielded results consistent with those obtained using multiple imputation (data not shown).

**Table 2:** Beta-binomial regression in participants with a cognitive dysfunction score > 20 (*n* = 1117)

Variable	Model 1		Model 2		Model 3	
	β (SE)	*p*	β (SE)	*p*	β (SE)	*p*
Occupational sitting (mean hours/day)	-0.012 (0.013)	0.34	-0.014 (0.013)	0.28	-0.011 (0.013)	0.39
Leisure-time sitting (mean hours/day)	0.026 (0.017)	0.12	0.025 (0.017)	0.14	0.022 (0.017)	0.19

Values are presented on the logit scale. Model 1 was adjusted for age, sex, and educational level. Model 2 was further adjusted for leisure-time MVPA, sleep duration, risky alcohol intake, and emotional health limitations. Model 3 was further mutually adjusted for occupational and leisure-time sitting

## Discussion

In this study, we investigated the associations between domain-specific sitting and cognitive dysfunction in middle-aged Swedish adults. Higher amounts of occupational sitting were associated with lower odds of reporting cognitive dysfunction, whereas higher amounts of leisure-time sitting were associated with higher odds. These associations persisted after adjustment for established factors relevant to cognitive health, including educational level, MVPA at leisure-time, sleep duration, risky alcohol intake, and emotional health limitations, and remained after mutually adjusting for occupational- and leisure-time sitting. Together, our findings suggest that the context in which sitting occurs may be important for understanding its relationship with subjective cognitive health.

The knowledge regarding the associations between domain-specific sitting and cognitive functions is still scarce. Wanders et al. [11] studied adults with a median age of 61 years and observed higher cognitive function among participants with the highest total sitting time. However, further analyses showed that work-related sedentary behavior and non-occupational computer time were associated with better cognitive function, whereas total leisure-related sedentary behavior showed no significant associations. Consistent with our findings, these associations remained significant after adjustment for potential confounders, including alcohol habits and depression [11]. Similarly, Hayat et al. [12] investigated physical inactivity across work and leisure domains and observed that occupational inactivity was related with lower risk of poor cognitive function in both cross-sectional and prospective analyses. However, leisure-time inactivity was related to a higher risk of poor cognitive function, but only in their cross-sectional analyses. Together, these findings support that the context and nature of activities performed while sitting may be important for cognitive health.

One important distinction between previous studies and the present study concerns how sedentary behavior has been measured. Previous studies have primarily relied on self-reported sedentary behavior or physical inactivity across different domains [11, 12]. Self-reported measures, especially single-items questions, are known to underestimate sitting time compared with device-based assessments [46]. Hence, the need for studies using accelerometer-based measurements of sedentary behavior has been emphasized [47]. By combining thigh-worn accelerometer data with work diaries, the present study provides a more precise assessment of both the duration and domain of sitting, specifically distinguishing between occupational and leisure-time sitting. However, despite this methodological strength, we were unable to determine the specific activities performed while sitting or the level of cognitive stimulation associated with those activities. It has previously been hypothesized that the type of other activity you do while sitting may be of greater importance for cognitive health rather than the total sitting time alone [6, 48]. In a recently published study, time spent in mentally active sedentary behavior was associated with a reduced risk of developing dementia [49]. In older adults, it has also been shown that more cognitively active sedentary behavior, such as using computers, is associated with better cognitive performance, while passive behaviors, such as watching television, are linked to poorer cognitive performance, both in cross-sectional and longitudinal analyses [50, 51]. Occupational and leisure-time sitting may involve different active or passive cognitive demands, making the associations between sedentary behavior and cognitive dysfunction potentially domain-specific. However, how different levels of cognitively active or passive sedentary behaviors are distributed during work- and leisure-time in middle-aged individuals and how it is associated with cognitive functions over the course of life need to be further studied, particularly during the retirement transition [52].

Previous research has suggested that MVPA to some extent may counteract some negative effects of a high level of sedentary behavior on cognitive outcomes in older adults, and that studies examining both factors simultaneously are needed [48]. In the present study, the associations of both occupational and leisure-time sitting with cognitive dysfunction remained significant after adjustment for leisure-time MVPA. Of note, we observed a relatively high amount of leisure-time MVPA in our study population. This is consistent with other studies, reporting 74–85 min of MVPA per day, when using thigh-worn accelerometers with similar processing methods as in our study [17, 53, 54]. These high levels of MVPA might be due to short burst of activities being included in the processing methods of data from the thigh-worn accelerometers. It may also be due to a healthy participant bias. For comparison, activity levels measured with the hip-worn ActiGraph accelerometer in a larger SCAPIS analysis of more than 27 000 participants have shown approximately 48 min per day of moderate-intensity physical activity, with only about 1 min per day in vigorous physical activity [55]. The high levels of MVPA in our study population may have created a ceiling effect, potentially explaining why MVPA did not alter the associations between domain-specific sitting and cognitive dysfunction.

The associations between sedentary behavior, sleep duration and brain health are complex and remain incompletely understood. Studies applying isotemporal substitution models have shown that replacing sedentary time with MVPA was related to a better cognitive function in adults over the age of 60 years, especially among those with no more than 7 h of sleep per night [56]. Similar results were observed by Yiallourou et al. [57] where the benefits on the risk of dementia of replacing sleep with inactivity or MVPA were different depending on habitual sleep durations (< 6 h or 6–9 h). Replacing sleep with light-intensity physical activity, however, gave similar results on the risk of dementia regardless of habitual sleep duration. In the present study, the associations between domain-specific sitting and cognitive dysfunction remained significant after adjusting for accelerometer-estimated sleep duration. Future studies should further investigate how combinations and interactions of sleep, physical activity and sitting behaviors across different domains, as well as during the 24-hour window, relate to cognitive health.

We also adjusted for several other potential confounding factors relevant for both sedentary behavior and cognitive functions. For example, depression and excessive alcohol intake have previously been identified as modifiable risk factors for dementia [4]. As discussed in Maasakkers et al., risk factors that may influence both sedentary behavior and cognitive dysfunction may cluster differently depending on the type and domain of sedentary behavior [48]. For example, social engagement, alcohol consumption or dietary intake may vary across sedentary contexts and could therefore differentially affect associations with cognitive functions [48]. In the present analyses, however, adjustment for risky alcohol intake and emotional health limitations did not alter the observed associations for either occupational or leisure-time sitting. Nevertheless, as we did not have any information on depression or anxiety, we relied on only two questions addressing emotional health limitations, which may have limited our ability to fully adjust for this potential confounding.

Our results add to the growing body of research on the health effects of domain-specific physical behavior, including physical activity and sedentary behavior. Whereas the health effects of leisure-time physical activity are well-known, research on occupational physical activity indicates that this may not be as beneficial for health. Higher levels of occupational physical activity have, for example, been associated with increased cardiovascular strain and adverse vascular effects [58]. Further, in a recently published meta-analysis, it was observed that physical activity performed during leisure-time and during household activities was associated with a reduced risk of all-cause dementia, whereas occupational and commuting physical activity was associated with an increased risk of all-cause dementia [59]. These findings suggest that the health effects of physical activity depend not only on how much activity that is performed, but also on the setting and circumstances in which it occurs. The evidence on this so-called physical activity paradox is however still limited, and more high-quality research is needed, focusing on e.g. better measurement methods of all parts of the physical behavior [58].

### Strengths and limitations

A key strength of this study is the thigh accelerometer-based measurements combined with the work diaries, which make it possible to analyze all physical behaviors during both work- and leisure-time. Further, this population-based study involves randomly selected men and women from the general population, enhancing the generalizability of the findings. In our models, we adjusted for several potential confounders of relevance for cognitive health and sedentary behavior. The consistency of the results between the multiple imputation and complete-case analyses strengthens the robustness of our findings.

The outcome measure in this study was subjective cognitive dysfunction and not objectively measured cognitive function based on e.g. computerized tests. Subjective measures may reflect not only objective cognitive performance, but also factors such as emotional health, stress and individual awareness of cognitive changes. The use of this particular subjective measure may also have limited the aspects of cognitive function captured in the present study, and different associations may have been observed if another measure of cognitive function had been used. However, the direction and magnitude of any such effect are difficult to determine. As previously mentioned, subjective cognitive decline has shown associations with future development of dementia and mild cognitive impairment [3].

The study population was middle-aged adults, with many participants reporting no or a low rate of cognitive dysfunction (Fig. 2). Also, the study population likely included few individuals with any intellectual disabilities. To the best of our knowledge, no established clinical cut-off value exists for the CDQ that can be used for diagnosing an individual with cognitive dysfunction. The availability of such thresholds could have facilitated interpretation of the clinical relevance of the results. More research using this scale in clinical populations is thus needed. Further, we did not have access to coded data regarding the participants’ current profession and could therefore not adjust our analyses for occupational characteristics and cognitive complexity, which may be of importance for cognitive health. For example, Rizvi et al. observed associations between high self-reported occupational sitting with higher cognitive functions, but these associations were partially explained by a combination of higher occupational cognitive complexity and educational level [60]. Further, in a previous multicohort study on over 100 000 participants, it was observed that individuals with cognitively stimulating jobs had a lower risk of developing dementia at a higher age, compared to individuals with less cognitively stimulating jobs, also when adjusting for other risk factors including physical inactivity [61]. However, other population-based cohorts have not observed such associations [62]. Future studies should carefully investigate the impact of different occupational belongings and characteristics in relation to sitting time and cognitive health. Further, the role of socioeconomic position could be more extensively explored. In our analysis, we adjusted for educational level, which represents one dimension of socioeconomic position. Other aspects include living conditions and economic and social resources. Future research should therefore examine a broader range of socioeconomic indicators.

Another potential limitation is the low prevalence of individuals with high CDQ scores, which may explain the lack of an association between sitting time and the severity of cognitive dysfunction.

Lastly, as this study is a cross-sectional study, we cannot draw any conclusions on causality. For example, it could be that those with sedentary leisure-time activities develop cognitive dysfunction to a higher extent, or that those with cognitive dysfunction are more prone to have a sedentary leisure-time. Longitudinal studies are therefore needed, to further explore the direction of causality.

## Conclusions

In middle-aged adults, occupational sitting was associated with lower odds, while leisure-time sitting was associated with higher odds, of reporting cognitive dysfunction. These associations were independent of educational level, leisure-time moderate- to-vigorous physical activity, sleep, risky alcohol intake and emotional health. However, neither occupational nor leisure-time sitting was related to the severity of symptoms among those already affected. Our results thus suggest that sitting may be more associated with the occurrence of cognitive dysfunction rather than its severity. Future research should explore these associations further using accelerometer-based measurements across different behavioral domains in studies using longitudinal designs. Additionally, the type of activity that is being performed while sitting and how it might differ in different domains needs further investigation.

## Data Availability

The datasets generated and analyzed during the current study are not publicly available due to the sensitive personal nature of the data. Information on procedures for data access, in compliance with Swedish legislation, can be obtained by contacting the corresponding author or the study organization (www.scapis.org).

## Abbreviations

CDQ: Cognitive Dysfunction Questionnaire
CI: Confidence Interval
IQR: Interquartile Range
MVPA: Moderate- to Vigorous Physical Activity
OR: Odds Ratio
SCAPIS: Swedish CArdioPulmonary bioImage Study
